# Deliberate Switching of Single Photochromic Triads

**DOI:** 10.1038/srep41739

**Published:** 2017-01-31

**Authors:** Johannes Maier, Martti Pärs, Tina Weller, Mukundan Thelakkat, Jürgen Köhler

**Affiliations:** 1 Experimental Physics IV, University of Bayreuth, 95440 Bayreuth, Germany; 2 Applied Functional Polymers, University of Bayreuth, 95440 Bayreuth, Germany

## Abstract

Photochromic molecules can be reversibly converted between two bistable conformations by light, and are considered as promising building blocks in novel macromolecular structures for sensing and imaging techniques. We have studied individual molecular triads consisting of two strong fluorophores (perylene bisimide) that are covalently linked via a photochromic unit (dithienylcyclopentene) and distinguished between deliberate switching and spontaneous blinking. It was verified that the probability for observing deliberate light-induced switching of a single triad (rather than stochastic blinking) amounts to 0.8 ± 0.1. In a few exceptional cases this probability can exceed 0.95. These numbers are sufficiently large for application in sensitive biosensing, and super-resolution imaging. This opens the possibility to develop devices that can be controlled by an external optical stimulus on a truly molecular length scale.

During the continuous strive for miniaturization of electronic devices, the commonly used lithography technologies have reached their limitations. As an alternative strategy for the development of optoelectronic elements, recent activities have focused on photochromic molecules, which are highly interesting for optical data storage, biosensing, and super-resolution fluorescence imaging^1^,^2^,^3^,^4^,^5^,^6^,^7^,^8^,^9^. Such molecules can be reversibly transformed between two bistable forms by light, thereby changing their geometrical and electronic properties. Photochromic systems based on cis-trans isomerisation (stilbenes, azobenzenes)^10^,^11^ or photocyclization reactions (fulgides, diarylalkenes, azulenes, spiropyrans)^6^,^12^,^13^,^14^,^15^,^16^ have been studied extensively in the past. However, besides fascinating results also severe problems concerning weak contrast between the signals registered from the two states, low fatigue resistance, and/or crosstalk between read-out and switching have been reported. It turned out that photochromic molecules of the dithienylcyclopentene (DCP) type feature both a high fatigue resistance and a high photoreaction quantum yield, making this a promising class of molecules for the development of molecular photoswitches^17^,^18^,^19^. The DCP unit undergoes a photocyclization reaction from the open to the closed form upon irradiation with light in the UV spectral range (280 nm–350 nm) and a photocycloreversion reaction upon irradiation with light in the visible spectral range (450 nm–650 nm). In recent work, it has been demonstrated that it is possible to modulate the electrical and/or optical properties of molecular dyads, organic transistors, molecular wires, or nanoparticle networks as a function of the light-induced alteration of the state of the DCP^20^,^21^,^22^,^23^,^24^,^25^.

In a series of publications we have studied quantitatively the optical switching behaviour of ensembles of molecular triads consisting of two perylene bisimide (PBI) units that are covalently linked to a 1,2-bis(2-methyl-5-phenyl-3-thienyl)-perfluorocyclopentene (DCP) unit, using a phenyl bridge^26^,^27^,^28^,^29^. In this study, we employ a new triad in which the bridge is a biphenyl unit (see inset on the left hand side of Fig. 1). The absorption spectrum of the closed form of the DCP overlaps strongly with the emission spectrum of the PBI units giving rise to resonant energy transfer. This can be exploited to modulate the fluorescence intensity from the perylene subunits with high contrast ratio as a function of the isomeric state of the DCP, see scheme on the right hand side of Fig. 1. The design strategy of this triad, PBI-DCP-PBI, combines the favourable properties of the photoswitchable DCP units - high photoreaction quantum yields (only about 100 photons per molecule are needed for the photocyclization, and about 2000 photons per molecule for the photocycloreversion reaction, respectively^28^), and high fatigue resistance - with the superior fluorescence properties of the perylene derivatives^1^,^30^,^31^,^32^. In particular the involvement of the strong fluorophore PBI is favourable for combining these approaches with single-molecule techniques which might open new avenues for biosensing with ultimate sensitivity, ultra-high density optical data storage (one bit - one molecule), or super-resolution fluorescence imaging^33^,^34^,^35^,^36^,^37^.

On/off switching of the fluorescence intensity of a chromophore attached to a DCP unit upon irradiation with UV and visible light has already been claimed for single molecules in the past^38^,^39^,^40^,^41^. However, the data did not allow to distinguish unambiguously between deliberate light-induced switching of the fluorescence intensity and unwanted, yet in single-molecule spectroscopy unavoidable, stochastic blinking processes. The latter, also termed fluorescence intermittency, refers to a random alternation of the emission between emitting (*on*) and non-emitting (*off*) periods^42^,^43^,^44^, where the bright and dark periods cover essentially all experimentally accessible time scales. What could be established in the aforementioned experiments was an increase of the on/off toggling rate of single photochromic compounds upon increasing the UV illumination intensity^40^.

Obviously, the general problem that is faced with photochromic systems when going to the single-molecule regime is how to distinguish between deliberate switching and spontaneous blinking. Or more explicitely for the PBI-DCP-PBI triads: What is the conditional probability that the photochromic DCP unit underwent a conformational change given that a change of the fluorescence intensity from the PBI has been registered? In statistics this conditional probability is referred to as precision or positive predictive value (ppv). Here we present a detailed statistical analysis of the fluorescence switching of single PBI-DCP-PBI triads. In order to collect a sufficient amount of photons from the triads during the various illumination periods^45^, the PBI chromophores are connected to the DCP unit via a biphenyl bridge, which slows down the energy transfer from the PBI to the DCP. The photoreactions are further slowed down by cooling the samples to 10 K, which, in addition, inhibits undesired irreversible photobleaching. Both measures favour long observation times enabling several hundreds of illumination cycles for each individual triad without bleaching. This allows us also to perform several reference experiments on each individual triad. Having this information at hand, we find for the individual PBI-DCP-PBI triads a distribution for the ppv values that is centred around a mean of 0.8. In other words, if a change of the fluorescence intensity of the PBI upon appropriate irradiation of a single triad is registered, then this is for 80% of the cases due to a light-induced conformational change of the photochromic unit.

## Results

In order to locate the individual triads the sample was excited at 488 nm and a fluorescence image was recorded using a low-temperature widefield microscope. Subsequently, a single triad was selected from the image and the microscope was switched to confocal mode. From our experiments we find that only a fraction of about 30% of the investigated triads showed reversible switching between a high- and a low-fluorescent state upon illumination with UV and visible radiation. This is ascribed to the fact that the DCP units can take two conformations, parallel and antiparallel, and that only the antiparallel conformation can undergo the photocyclization reaction^46^. In the following we will focus on those triads that allowed for a light-induced modulation of the fluorescence intensity.

**Figure 2:**
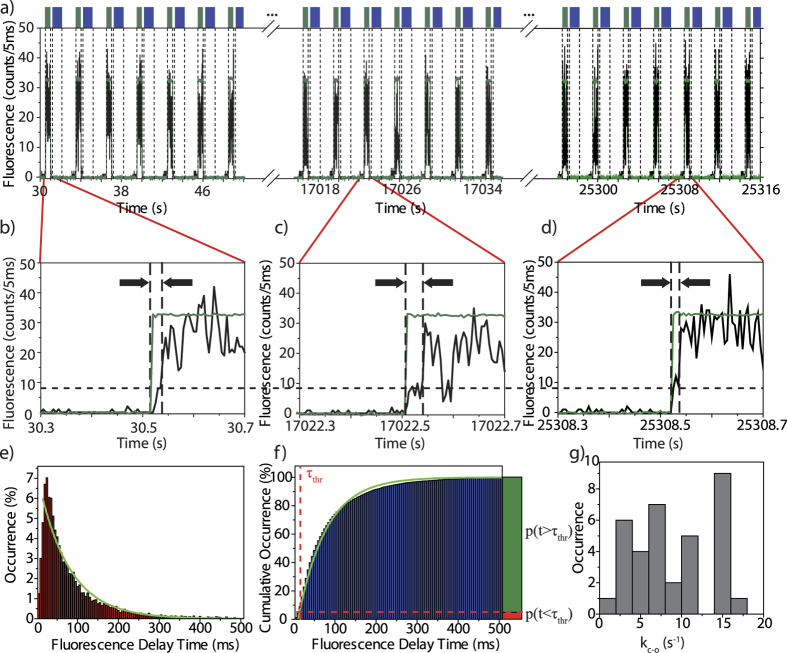
(**a**) Cut-outs of the fluorescence response (black line) of a single triad as a function of the illumination with radiation at 325 nm (blue, I_325_ = 1.78 W/cm^2^) and 488 nm (green, I_488_ = 120 W/cm^2^) as indicated on top of the panel. The emission is sampled with a dwell time of 5 ms. Rising edges of individual switching cycles are shown in (**b**–**d**) on an expanded scale, where the temporal profile of the 488 nm illumination is given by the green line. The horizontal dashed line indicates the background level. (**e**) Relative and (**f**) cumulative distribution of the delay times for 8800 illumination sequences. The green lines correspond to an exponential decay (**e**), or asymptotic exponential growth (**f**), respectively. The vertical dashed line in (**f**) indicates the temporal threshold τ_thr_ that corresponds to twice the dwell time thereby setting the experimental time resolution. The horizontal dashed line separates the probabilities p(t < τ_thr_) and p(t > τ_thr_) at the threshold, visualised by the red and green bars on the right hand side. (**i**) Histogram of the ring-opening rate k_c-o_ = (τ_ON_)^−1^ for 13 individual triads based on experiments with 400 illumination sequences per triad.

In the first experiment we verify the light-induced switching of a single triad between a low- and a high-fluorescent state, and test the fatigue resistance. Therefore, a single triad is illuminated for 1 s at 325 nm with a fixed intensity of 1.78 W/cm^2^. This initialises the triad in a state with a closed DCP unit, corresponding to the state with reduced fluorescence intensity from the PBI units^26^,^40^. Then, after a waiting time of 1.3 s without any illumination the probe laser at 488 nm is switched on for 0.5 s. Radiation at this wavelength serves for both, conversion of the DCP to the open state and probing the fluorescence of the PBI units. After another waiting time of 0.2 s without illumination the whole illumination sequence is repeated 8800 times corresponding to a total duration of the experiment of 26400 s (7 h 20 min). It is worth noting that the end of the experiment after more than 7 hours was determined by the experimentalist and not due to a deterioration of the triad. During the experiment the fluorescence from the triad is sampled with a dwell time of 5 ms. The result of this experiment is exemplified in Fig. 2a for three cut-outs of 20 s duration each from the full data set. The illumination sequences are indicated at the top of Fig. 2a by the coloured bars. Expanded views of the rising edges of the fluorescence intensity after switching on the probe laser are shown in Fig. 2b–d and reveal a delay of some ten milliseconds between the onset of the laser and the raise of the fluorescence. For the 8800 repetitively carried out switching sequences the distribution of these times is shown in Fig. 2e and follows an exponential decay (green line, Fig. 2e). Given a temporal resolution of two times the dwell time, i.e. 10 ms, we note that the fraction of delay times longer than 10 ms amounts to about 95%.

In order to quantify our observations we represent the histogram of the delay times as a cumulative distribution function (CDF) defined as


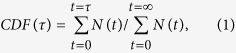


where N(t) denotes the number of events with a delay time *t*. The CDF(τ) corresponds directly to the probability to observe a change of the fluorescence intensity during the time interval 0 < *t* ≤ τ. The respective CDF for the histogram shown in Fig. 2e is displayed in Fig. 2f.

It can be fitted by an asymptotic exponential growth of the type 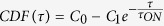 (green line, Fig. 2f), where C_0_ and C_1_ represent an offset and an amplitude, respectively, and τ_ON_ represents the average delay time that will be referred to as *switch-on-time* hereafter. For the example shown we find τ_ON_ = 75.7 ms. According to the model developed in ref. 28, the switch-on-time can be associated with the close-to-open conversion rate k_c−o_ via k_c−o_ = (τ_ON_)^−1^. The distribution of this rate is shown in Fig. 2g for 13 individual triads. For these experiments each single triad was exposed to 400 consecutive illumination sequences at similar excitation conditions as detailed above. The rates found for k_c−o_ range from 1.2 s^−1^ to 48.8 s^−1^ with a mean value of 10.7 s^−1^. This variation might be caused by differences in the quantum yield of the ring opening reaction among the individual triads due to different local environments, but more probably it reflects the different out-of-plane orientations of the transition-dipole moments of the individual triads with respect to the polarization of the incident light field, resulting in variations of the effective excitation intensity.

**Figure 3:**
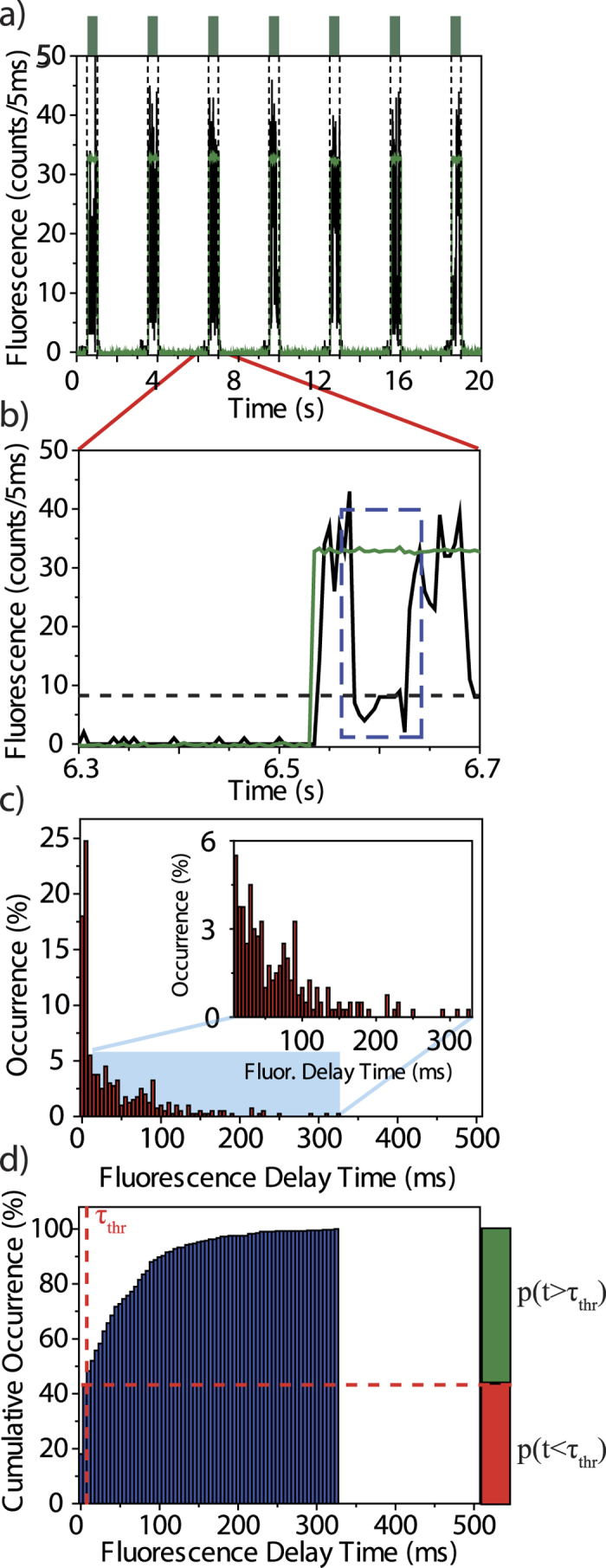
Control experiment on the same triad as used forFig. 2 without 325 nm illumination. (**a**) Cut-out of the fluorescence response (black line) as a function of the illumination at 488 nm (green) as indicated on top of the panel. The emission was sampled with a dwell time of 5 ms. (**b**) Single illumination cycle on an expanded scale. The green line corresponds to the temporal profile of the 488 nm illumination. The horizontal dashed line indicates the background level. (**c**) Relative distribution of the delay times for 400 illumination sequences. The inset shows the blue shaded area on an expanded scale. (**d**) Cumulative distribution function of the delay times for an excitation intensity of 120 W/cm^2^ at 488 nm. The vertical dashed line indicates the temporal threshold τ_thr_ that corresponds to the experimental time resolution. The horizontal dashed line separates the probabilities p(t < τ_thr_) and p(t > τ_thr_) at the threshold, red and green bars, respectively.

In order to verify that we deal indeed with photochromic switching of an individual triad rather than blinking, we performed for each triad under study a control experiment without exposing the molecule to the 325 nm beam and applied only the radiation at 488 nm. The corresponding data for the triad that has been used for Fig. 2 are shown in Fig. 3. Now the onset of the fluorescence follows directly the modulation of the intensity of the excitation laser without delay, see Fig. 3b. This is also evident from the histogram of the delay times, Fig. 3c whose entries accumulate around time bins corresponding to time delays of 5 to 10 ms. Since our data acquisition runs with 5 ms sampling time, this reflects simply the achievable temporal resolution of the experiment, i.e. two time bins. The corresponding CDF is displayed in Fig. 3d and is not compatible with the type of exponential function used before. However, closer inspection of the fluorescence time traces reveals also the typical blinking of single molecules, see for example the blue dashed box in Fig. 3b after about 6.55 s. If such blinking events occur within the first 10 ms after switching on the probe beam these will be registered as a finite “delay time”, which explains the entries in the range between 10 ms and 300 ms in the histogram Fig. 3c. For the two-beam experiments such blinking events contribute as “false positives” (*vide infra*) to the histogram of the delay times in Fig. 2e.

**Figure 4:**
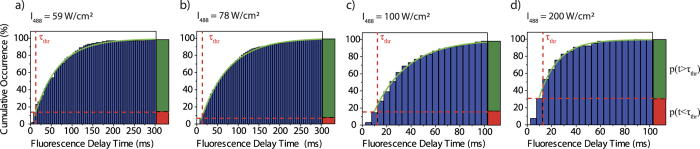
Cumulative distribution functions of the fluorescence delay times as a function of the 488 nm excitation intensity. (**a**) 59 W/cm^2^; (**b**) 78 W/cm^2^; (**c**) 100 W/cm^2^; (**d**) 200 W/cm^2^. All distributions correspond to experiments with 400 consecutive illumination sequences. The data have been sampled with dwell times of 10 ms (**a,b**) and 5 ms (**c,d**), respectively. Note the different scales in (**a,b**) versus (**c,d**). The green lines correspond to an asymptotic exponential growth, whereas the vertical dashed lines indicate the temporal threshold τ_thr_ that corresponds to twice the dwell time thereby setting the experimental time resolution. The horizontal dashed lines separate the probabilities p(t < τ_thr_) and p(t > τ_thr_) at the threshold, visualised by the red and green bars on the right hand side. The experiments were carried out at 10 K on the same single triad.

Next we studied the switching behaviour of an individual triad as a function of the intensity of the probe beam at 488 nm. Therefore this intensity was varied between 59 W/cm^2^ and 200 W/cm^2^, and in each experiment the single triad was exposed to 400 illumination sequences. The resulting CDFs are shown in Fig. 4 and can all be described by an asymptotic exponential growth of the form 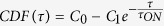. For the corresponding switch-on-times we find τ_ON_ = 57.1 ms (59 W/cm^2^), 66.4 ms (78 W/cm^2^), 27.3 ms (100 W/cm^2^), and 15.2 ms (200 W/cm^2^), respectively, featuring a monotonic decrease for increasing the 488 nm intensity above 78 W/cm^2^.

## Discussion

For the two-beam experiment the DCP is initialised in the closed state (low PBI fluorescence yield) and illumination at 488 nm first converts the DCP to the open state before the PBI fluorescence with high emission yield is initiated. Generally, both the light-induced conversion of the photochromic unit and the emission of a photon from the PBI are quantum mechanical processes that obey Poissonian statistics. However, the typical time scale for the latter is in the nanosecond range which is beyond the temporal resolution of our experiment, and we can only follow the first process. As a consequence of the Poissonian statistics for the light-induced conformational change, the distribution for finding a delay time in the interval between τ and τ + dτ is given by an exponential decay. As can be seen from Fig. 2e the data are clearly in line with this expectation. Mathematically the distribution for the delay times corresponds to a probability density, and its integral, here presented as the CDF(τ), gives directly the probability for observing a switching event during the time interval [0, τ]. Hence, for the current situation the CDF is expected to have the form 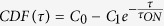 which is also corroborated by the data, see Figs 2f and 4. In contrast, for illumination at 488 nm only, the DCP unit is already preinitialised in the open state. This explains why, given the temporal resolution of the experiment, the onset of the fluorescence promptly follows the rising edge of the laser intensity.

Accordingly, the experimentally accessible readout values for the onset of the fluorescence are either “delay zero” or “finite delay”. However, due to the unavoidable blinking, see Fig. 3, a delayed onset cannot directly be associated with a deliberate switching of the photochromic unit, and we have to resort to the concept of conditional probabilities to quantify our results. Let S (S for switching) refer to the event that the triad is initially prepared in the closed state and that it undergoes a conformational change to the open state upon irradiation with 488 nm, and let 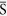 refer to the event that the triad does not change its intial conformational state. Analogously, let “+” refer to the event “registration of a finite delay”, and “−” denote the event “registration of delay zero”, i.e. a prompt onset of the fluorescence. Then, for example, p(+|S) refers to the conditional probability that we register a finite delay time given that the DCP underwent a conformational change from the closed to the open state (“true positive”). Likewise, 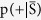 denotes the conditional probability that we register a finite delay time (for example due to blinking) under the condition that the DCP did not change its conformation (“false positive”). Defining a temporal threshold, τ_thr_, that takes our temporal resolution into account, allows us to read probabilities for registering specific events from a single molecule directly from the CDFs in Figs 2f and 4. For example, the green bar on the right hand side of Fig. 2f indicates the probability for registering finite delay times p(t > τ_thr_) irrespective whether the DCP underwent a conformational change or not. This represents to the total probability to register a finite delay time given as^47^ 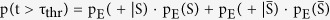, where p_E_(S) refers to the probability for a conformational change of the DCP from the closed to the open state, and 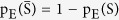 refers to the probability that the DCP does not change its conformation. The index E refers to the experimental situation with two laser beams. For the CDF from the control experiment displayed in Fig. 3d the situation is slightly different. From the design of the control experiment we know by definition that the DCP unit will not undergo a light-induced change of its conformation. Hence, here p(t > τ_thr_) corresponds to the conditional probability that we register a finite delay time under the condition that the molecule did not change its conformational state, i.e. 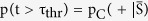. Here the index *C* refers to the control experiment. However, the interesting statistical parameter is the positive predictive value (ppv), p_E_(S|+), i.e. the probability that the triad indeed underwent a conformational change given that we have registered a finite delay. Using the expression for the total probability p_E_(+) and applying Bayes law^47^ this yields


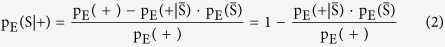


The conditional probability 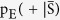 can be read from the CDF in Fig. 3f using the approximation 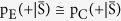, and a quantitative estimate for 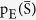 can be obtained from a reference experiment that concerns solely the blinking behaviour of the triad under study, see SI. Using the figures obtained from the experiments (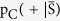 = 0.57, p_E_(+) = 0.96, and 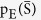 = 0.50) this yields a positive predictive value of 0.70.

**Figure 5:**
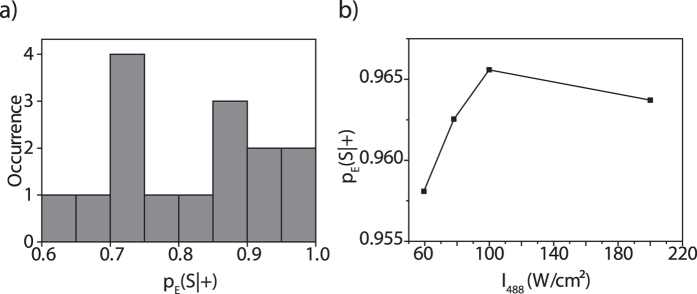
(**a**) Distribution of the positive predictive values p_E_(S|+) for 15 individual PBI-DCP-PBI triads, initialised with I_325_ = 1.78 W/cm^2^, and probed with I488 = 200 W/cm^2^. (**b**) Positive predictive value p_E_(S|+) for one selected individual PBI-DCP-PBI triad as a function of the intensity of the probe beam.

In Fig. 5a the distribution of the ppv is shown for 15 single triads that were studied for 400 illumination sequences each, using I_325_ = 1.78 W/cm^2^ and I_488_ = 200 W/cm^2^. The obtained ppvs range from 0.62 to 0.97 centred at 0.81 ± 0.11 (mean ± sdev). For one of the triads that featured a relatively large ppv of 0.964, we studied as well the ppv as a function of the intensity of the probe beam, Fig. 5b. This reveals only a weak variation of this parameter between 0.958 and 0.965 for changing the intensity I_488_ between 60 W/cm^2^ and 200 W/cm^2^, featuring a peak value of 0.965 for I_488_ = 100 W/cm^2^.

We have shown that the particular photochromic triad system features excellent fatigue resistance with high contrast between the detected signals in the two light induced conformations. Exploiting the high fluorescence yield of the perylene based chromophores attached to the photochromic unit allows to observe photochromic switching at the single molecule level. We verified that the probability to observe deliberate light-induced switching processes rather than stochastic blinking processes from a single triad is on average about 80%, and can exceed 95% in a few exceptional cases. These numbers are sufficiently large for application in sensitive biosensing, and super-resolution imaging^45^. However, for optical data storage these numbers are far too small, and further research is required to elucidate the key parameters that finally determine the actual value of the positive predictive value.

## Materials and Methods

### Sample preparation

The triad PBI-DCP-PBI was synthesized in a multi-step reaction starting from the commercially available 2-methylthiophene as shown in SI. For the photoswitching experiments the triads were immobilized in a Poly(methyl methacrylate) (PMMA) matrix. Therefore the triads were dissolved in chloroform at a concentration of 4.4 · 10^−10^ mol/l. This solution was mixed at equal fractions with a second chloroform solution that contained PMMA (10 mg of PMMA per 1 ml of chloroform). About 50 μl of this mixture was then spincoated on a microscope coverslip producing a polymer film with a thickness of about 200 nm. This sample was mounted in a helium flow microscope cryostat (KONTI-Cryostat, CryoVac). All experiments were carried out at 10 K.

### Photoconversion experiments

As light sources we used a solid state laser diode (Cyan Laser, Spectra Physics) and a Helium-Cadmium laser (Kimmon, IK3201R-F), providing radiation at 488 nm and 325 nm, respectively. The light at 488 nm is reflected by a dichroic mirror DM (525 DCXRU, AHF) towards a long distance objective (M Plan APO (50x, NA = 0.55, infinitiy corrected), Edmund Optics) and focused onto the sample through the front window of the cryostat and a home build solid immersion lens (SIL, Edmund Optics) mounted inside the cryostat^48^,^49^,^50^. The radiation at 325 nm is focused by a lens (f = 50 mm) through the back window of the cryostat to a spot with a diameter of 19.6 μm on the sample. The fluorescence from the sample is collected by the same objective system, transmitted through the dichroic mirror and passes a bandpass filter (AHF, HQ 525 BP, transmission 525–725 nm, AHF) to suppress residual stray light.

For locating the individual triads the optics was operated in widefield mode for imaging and the signal is detected with a back-illuminated EMCCD camera (iXon DV877ECS, Andor). Subsequently the optics was converted to confocal mode for addressing individual triads and an avalanche photodiode (MPD-5CTC, PicoQuant) served as a detector. Sequential exposure of the sample with the appropriate radiation is accomplished by using electromechanical shutters that could be opened and closed with ~5 ms temporal accuracy. For synchronizing the timing between the detected fluorescence signal and probe beam at 488 nm, the output from the laser diode was monitored with a photodiode (S2387, Hamamatsu).

## Additional Information

**How to cite this article**: Maier, J. *et al*. Deliberate Switching of Single Photochromic Triads. *Sci. Rep.* **7**, 41739; doi: 10.1038/srep41739 (2017).

**Publisher's note:** Springer Nature remains neutral with regard to jurisdictional claims in published maps and institutional affiliations.

## Supplementary Material

Supporting Information

## Figures and Tables

**Figure 1:**
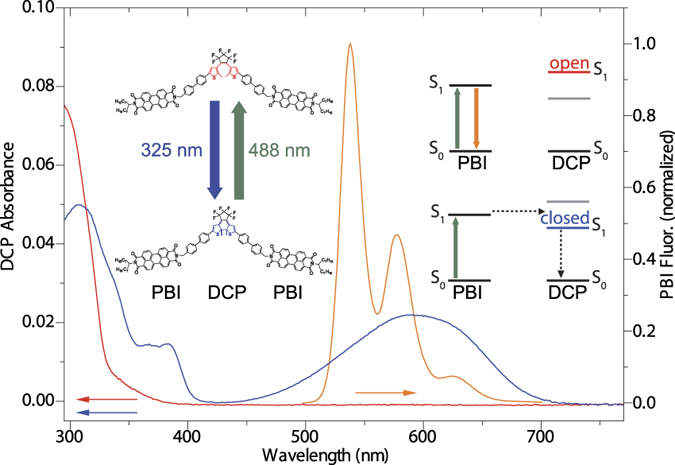
Ensemble absorption spectra of DCP in its closed (blue) and open (red) form, overlaid with the ensemble emission spectrum of PBI (orange), all for solutions in toluene. The inset on the left hand side shows the molecular structure of the triad PBI-DCP-PBI in its two conformations. The DCP unit undergoes a photocyclization reaction from the open (top) to the closed form (bottom) upon irradiation with light in the UV spectral range (blue arrow) and a photocycloreversion reaction upon irradiaton with visible light (green arrow). The inset on the right hand side shows the energy level structure of the lowest electronically excited states of the PBI and the DCP allowing for resonant energy transfer from the PBI to the closed form of the DCP leading to a reduction of the fluorescence intensity from the PBI.
