# Supplementary material for: Deliberate Switching of Single Photochromic Triads
Source: Sci Rep. 2017 Jan 31;7:41739. doi: 10.1038/srep41739 (PMC5282491; doi:10.1038/srep41739)
Supplement: Supporting Information [file srep41739-s1.pdf]

Supporting Information for

Deliberate Switching of Single Photochromic Triads

Johannes Maier[1#], Martti Pärs[1#†], Tina Weller[2],
Mukundan Thelakkat[2], and Jürgen Köhler[1]*

[1] *Experimental Physics IV, University of Bayreuth, 95440 Bayreuth, Germany*

[2] *Applied Functional Polymers, University of Bayreuth, 95440 Bayreuth, Germany*

[†]Current address:

Institute of Physics, University of Tartu, W. Ostwaldi str. 1, 50411 Tartu, Estonia

[#]these authors contributed equally to this work

*Corresponding author: juergen.koehler@uni-bayreuth.de

Table of Contents

1. Chemical Synthesis
2. Blinking Experiment

1. Chemical Synthesis

All commercially available chemicals (Aldrich, Abcr) were used as received without further purification. Anhydrous solvents were purchased from Sigma Aldrich and Acros in sealed bottles over molecular sieve. Column chromatography was carried out on Silica gel 60M (0.040-0.063 mm particle size, 230-400 mesh ASTM) purchased from Machery-Nagel. ^{1}H-NMR spectra were recorded on a Bruker AC 300 spectrometer with deuterated chloroform as solvent. Infrared spectra were recorded as ATR on a Perkin Elmer Spectrum 100 FTIR spectrometer. Mass spectrometry was performed on a Finnigan MAT 8500 EI-MS (ionization energy 70 eV). MALDI-TOF MS was conducted on a Bruker Reflex III using using *trans*-2-(3-(4-tert-butylphenyl)-2-methyl-2-propenylidene)malononitrile (DCTB) as matrix and silver trifluoroacetate (AgTFA) as cationizing salt. Solutions from the triads in chloroform (0.01 mg/μL), matrix in chloroform (0.01 mg/μL) and salt in tetrahydrofuran (0.01 mg/μL) were mixed in the ratio 5:20:1 (v:v:v) and spotted onto the MALDI plate.

The triad PBI-DCP-PBI was synthesized in a multi-step reaction starting from the commercially available 2-methylthiophene as shown in the scheme below. 3-bromo-2-methyl-5-(4,4,5,5-tetramethyl-1,3,2-dioxaborolan-2-yl)-thiophene **(2)** was synthesized according to the literature [1,2]. Following published procedures the reagents 4-bromo-1,1-biphenyl-4-carbaldehyde **(3)**[3] and *N*-(1-heptyloctyl)perylene-3,4:9,10-tetracarboxybisimide **(8)** [4] were synthesized.

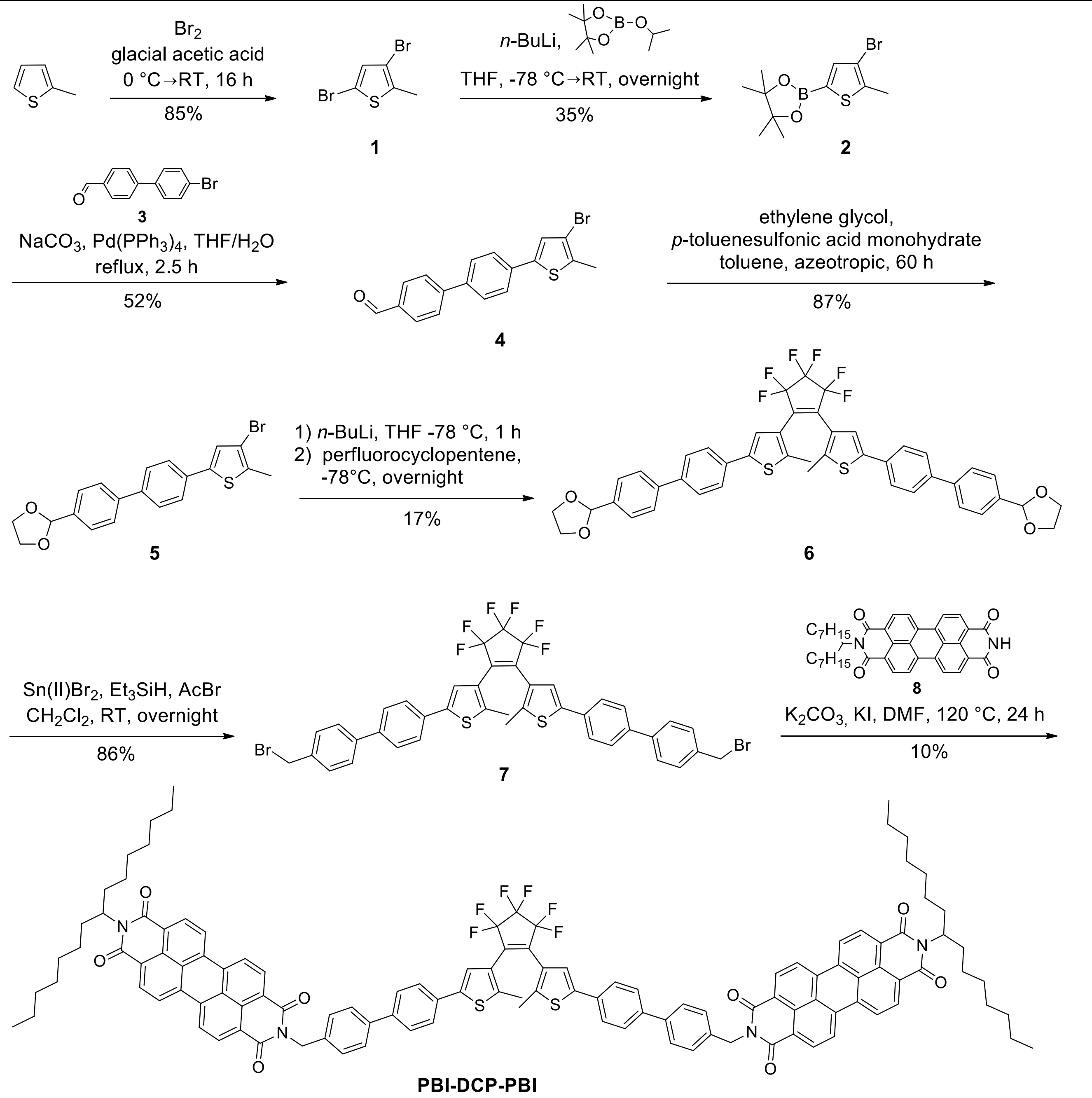

Supporting Scheme 1: Synthetic scheme for the PBI-DCP-PBI triad.

Synthesis of 3-Bromo-5-(4-formyl-1,1-biphenyl)-2-methylthiophene (4)

A mixture of 3-bromo-2-methyl-5-(4,4,5,5-tetramethyl-1,3,2-dioxaborolan-2-yl)-thiophene **(2)** (2.23 g, 7.36 mmol, 1.0 eq) in a biphasic system of 24 mL tetrahydrofuran and 18 mL aqueous solution of 20% $NaCO_3$ was degassed by argon purging for 1 h. To the reaction mixture 4-bromo-1,1-biphenyl-4-carbaldehyde **(3)** (2.60 g, 9.96 mmol, 1.4 eq) and

tetrakis(triphenylphosphine)-palladium(0) (0.49 g, 0.43 mmol, 0.06 eq) were added. The reaction mixture was stirred at 80 °C for 2.5 h while the mixture changed primarily its color to red followed by precipitation of a yellow solid. After cooling to room temperature, tetrahydrofuran was added until the yellow precipitate was dissolved. The organic layer was separated and washed with saturated aqueous solution of $NaHCO_3$. After drying over $MgSO_4$, the extract was filtered and the solvent was removed under reduced pressure. The crude material was purified using flash column chromatography (silica; DCM) and further purified by recrystallization from ethyl acetate. The crystals were separated and washed with a small amount of cold ethyl acetate and diethyl ether yielding 1.36g 3-bromo-5-(4-formyl-1,1-biphenyl)-2-methylthiophene **(4)** (3.81 mmol, 52%) as a pale yellow solid.

^{1}H-NMR (300 MHz, $CDCl_3$): δ = 10.06 (s, 1H), 7.97 (d, ^{3}J = 8.5 Hz, 2H), 7.77 (d, ^{3}J = 8.2 Hz, 2H), 7.68-7.60 (m, 4H), 7.19 (s, 1H), 2.44 (s, 3H) ppm. EI-MS (70 eV): *m/z* 358 [$(M+H)^+$]. IR (FT, ATR): $\tilde{\nu}$ = 1700, 1598, 1495, 1390, 1193, 1165, 1135, 1000, 839, 809, 795, 743, 702, 675 cm^{-1}.

Synthesis of 3-Bromo-5-(4-(2,5-dioxolanyl)-1,1-biphenyl)-2-methylthiophene (5)

A mixture of 3-bromo-5-(4-formyl-1,1-biphenyl)-2-methylthiophene **(4)** (1.14 g, 3.19 mmol, 1.0 eq), 1.96 mL ethylene glycol (35.1 mmol, 11 eq) and *p*-toluenesulfonic acid monohydrate (0.006 g, 0.032 mmol, 0.01 eq) was dissolved in 100 mL toluene. The solution was heated to 135 °C for 60 h using a Dean-Stark apparatus. After cooling to room temperature, the reaction mixture was poured into 100 mL aqueous saturated solution of $NaHCO_3$. The layers were separated and the aqueous layer was extracted with chloroform twice. The combined organic layers were washed alternately with aqueous saturated solution of $NaHCO_3$ and water twice. After drying over $MgSO_4$, filtration and removal of the solvent using rotary evaporation 1.12 g 3-bromo-5-(4-(2,5-dioxolanyl)-1,1-biphenyl)-2-methylthiophene **(5)** (2.79 mmol, 87%) were obtained as a pale brown solid.

^{1}H-NMR (300 MHz, $CDCl_3$): δ = 7.65-7.54 (m, 8H), 7.15 (s, 1H), 5.87 (s, 1H), 4.20-4.04 (m, 4H) ppm. EI-MS (70 eV): *m/z* 402 [$(M+H)^+$]. IR (FT, ATR): ṽ = 2957, 2879, 2729, 1699, 1597, 1497, 1196, 1174, 1077, 1001, 943, 808, 792, 772, 744, 663 cm^{-1}.

Synthesis of 1,2-Bis(2-methyl-5-(4-(2,5-dioxolanyl)-1-1-biphenyl)thiophen-3-yl) hexa-fluoro-cyclopentene (6)

3-Bromo-5-(4-(2,5-dioxolanyl)-1,1-biphenyl)-2-methylthiophene **(5)** (1.05 g, 2.62 mmol, 1.0 eq) was dissolved in 50 mL anhydrous tetrahydrofuran and cooled to -78 °C. The solution was treated slowly with 1.80 mL 1.6 M *n*-butyl lithium (2.88 mmol, 1.1 eq) and stirred for 1 h at -78 °C. After addition of 0.18 mL perfluorocyclopentene (2.88 mmol, 0.5 eq), the reaction mixture was stirred for 6 h at -78 °C and was slowly allowed to warm to room temperature overnight under stirring. To the solution 50 mL aqueous saturated solution of NH_4Cl was added and the layers were separated. The aqueous layer was extracted with diethyl ether three times and the combined organic layers were washed with aqueous saturated solution of $NaHCO_3$ three times. After drying over $MgSO_4$, the extract was filtered and the solvent was removed under reduced pressure. The brown residue was purified using flash column chromatography (silica; DCM) yielding a mixture of 1,2-bis-(2-methyl-5-(4-(2,5-dioxolanyl)-1-1-biphenyl)thiophen-3-yl-)hexafluorocyclopentene and the deprotected hexafluorocyclopentene derivative. The crude material, 0.30 mL ethylene glycol (5.36 mmol, 2.0 eq) and a catalytic amount of *p*-toluenesulfonic acid monohydrate was dissolved in 40 mL toluene. The reaction mixture was heated to 135 °C for 60 h using a Dean-Stark apparatus. After cooling to room temperature, the reaction mixture was poured into 20 mL aqueous saturated solution of $NaHCO_3$. The layers were separated and the aqueous layer was extracted with diethyl ether twice. The combined organic layers were washed alternately with aqueous saturated solution of $NaHCO_3$ and water twice. After drying over $MgSO_4$, filtration and removal of the solvent using rotary evaporation 0.36 g 1,2-bis(2-methyl-5-(4-(2,5-dioxolanyl)-1-1-biphenyl)thiophen-3-yl-)hexafluorocyclopentene **(6)** (0.441 mmol, 17%) was obtained as a blue solid.

^{1}H-NMR (300 MHz, $CDCl_3$): δ = 7.67-7.54 (m, 16H), 7.31 (s, 2H), 5.88 (s, 2H), 4.20-4.04 (m, 8H), 1.97 (s, 6H) ppm. EI-MS (70 eV): *m/z* 816 [$(M)^+$]. IR (FT, ATR): ṽ = 2962, 2880, 1388, 1337, 1271, 1190, 1135, 1112, 1081, 1054, 987, 942, 812, 740 cm^{-1}.

Synthesis of 1,2-Bis(2-methyl-5-(4-bromomethyl-1,1-biphenyl)thiophen-3-yl) hexa-fluorocyclo-pentene (7)

A mixture of 1,2-bis(2-methyl-5-(4-(2,5-dioxolanyl)-1-1-biphenyl)thiophen-3-yl-)hexa-fluorocyclopentene **(6)** (314 mg, 0.380 mmol, 1.0 eq) and tin(II)bromide (11.8 mg, 0.042 mmol, 0.11 eq) was suspended in 15 mL anhydrous dichloromethane. To the suspension 0.27 mL triethylsilane (1.20 mmol, 3.1 eq) were added followed by 0.13 mL acetyl bromide (1.69 mmol, 4.4 eq). The reaction mixture was stirred at room temperature overnight. After the addition of 40 mL phosphate buffer (pH 7), the layers were separated and the aqueous layer was extracted with dichloromethane three times. The combined organic layers were washed with aqueous saturated solution of $NaHCO_3$ and brine. After drying over $MgSO_4$, filtration and removal of the solvent using rotary evaporation, the oily residue was purified using flash column chromatography (silica; DCM/*i*Hex 2:1). The product was freeze-dried from benzene yielding 282 mg 1,2-bis(2-methyl-5-(4-bromomethyl-1,1-biphenyl)thiophen-3-yl)hexa-fluorocyclopentene **(7)** (0.328 mmol, 86%) as a blue solid.

^{1}H-NMR (300 MHz, $CDCl_3$): δ = 7.64-7.57 (m, 12H), 7.51-7.46 (m, 4H), 7.34 (s, 2H), 4.56 (s, 4H), 1.99 (s, 6H) ppm. EI-MS (70 eV): *m/z* 858 [$(M)^+$]. IR (FT, ATR): ṽ = 2982, 2911, 2866, 1501, 1469, 1436, 1400, 1337, 1269, 1229, 1190, 1102, 1054, 986, 895, 886, 808, 740, 728 cm^{-1}.

Synthesis of 1,2-Bis(2-methyl-5-(N-(pentadecan-8-yl)-perylene-3,4:9,10-tetracarboxy-bisimid)-1,1-biphenyl)thiophen-3-yl)hexafluorocyclopentene (*PBI-DCP-PBI*)

A mixture of 1,2-bis(2-methyl-5-(4-bromomethyl-1,1-biphenyl)thiophen-3-yl-)hexafluoro-cyclopentene **(7)** (94.0 mg, 0.109 mmol, 1.0 eq), *N*-(1-heptyloctyl)perylene-3,4:9,10-tetracarboxybisimide (360 mg, 0.599 mmol, 5.5 eq) **(8)**, potassium carbonate (1.16 g, 8.39 mmol, 30 eq) and a catalytic amount of potassium iodide were dissolved in 20 mL

anhydrous dimethylformamide. The reaction mixture was heated to 120 °C for 24 h. After cooling to room temperature, water was added and the aqueous layer was extracted with chloroform three times. The combined organic layers were washed with saturated aqueous solution of $NaHCO_3$ and dried over $MgSO_4$. The solvent was removed under reduced pressure and the crude product was purified using flash column chromatography (silica; 1% acetic acid in $CHCl_3$). Further purification by MPLC (silica; 1% acetic acid in $CHCl_3$) yielded 20 mg 1,2-bis(2-methyl-5-(*N*-(pentadecan-8-yl)-perylene-3,4:9,10-tetra-carboxybisimid)-1,1-bi-phenyl)thiophen-3-yl)hexa-fluorocyclopentene (0.011 mmol, 10%) as a dark red solid.

^{1}H-NMR (300 MHz, $CDCl_3$): δ = 8.73-8.49 (m, 16H, H-5), 7.70-7.49 (m, 16H, H-3), 7.29 (s, 2H, H-1), 5.44 (s, 4H, H-4), 5.25-5.12 (m, 2H, H-6), 2.32-2.18 (m, 4H, H-7), 1.97-1.78 (m, 4H, H-7), 1.94 (s, 6H, H-2), 1.39-1.16 (m, 40H, H-8), 0.81 (t, ^3J = 6.5 Hz, 12H, H-9) ppm. MS (MALDI): *m/z* calculated for $[M+Ag]^+$: 2003.64; found: 2003.81. IR (FT, ATR): $\tilde{\nu}$ = 2922, 2851, 1696, 1659, 1595, 1403, 1334, 1269, 1247, 1110, 986, 850, 809, 741 cm^{-1}. IR (FT, ATR): $\tilde{\nu}$ = 2982, 2911, 2866, 1501, 1469, 1436, 1400, 1337, 1269, 1229, 1190, 1102, 1054, 986, 895, 886, 808, 740, 728 cm^{-1}.

2. Blinking Experiment

For the reference experiments that address the blinking of individual triads, these were illuminated solely with the radiation at 488 nm. For analysing the blinking statistics of the triad used for figs.2,3 and fig.4a-c of the main text, the data from the control experiment were appended, which yields a fluorescence intensity trace of 200 s duration. An excerpt of this trace is shown in fig.S1a revealing the typical telegraph-like blinking of single molecules.

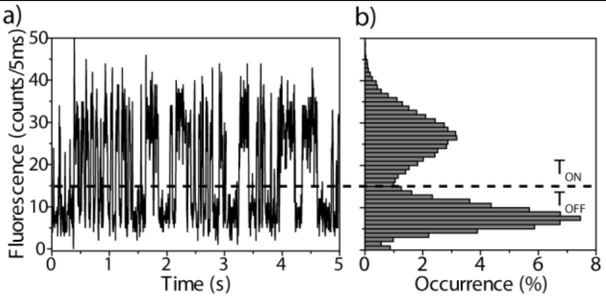

Supporting Figure 1: a) Excerpt of a fluorescence intensity trace from a single triad. The trace has been constructed from the fluorescence patches obtained in the control experiment described in fig.3a of the main text. Given the 400 illumination cycles of 0.5 s duration each, this amounts to a total length of the trace of 200 s. b) Distribution of the intensity levels for the full fluorescence intensity trace. The dashed line indicates the threshold used to discriminate between the ON and the OFF state

The distribution of the intensity levels of the full trace is shown in fig.S1b and features two peaks. The lower one has a maximum at 8 counts /5 ms (1600 counts / s) and corresponds to the background level (see also fig.2b-d,3b in the main text), whereas the higher one peaks at 27 counts /5 ms (5400 counts / s) and corresponds to the average intensity of the *ON* state. We used the minimum between these two peaks for discriminating the *ON* and the *OFF* levels (see dashed line in fig.S1). This yields about equal fractions for the residence times in those states, i.e. T_{OFF}/T = 50.4% and T_{ON}/T = 49.6%. Here T_{ON} (T_{OFF}) refers to the total accumulated residence time in the *ON* (*OFF*) level, and T to the total time of the experiment.

For the reference experiments used to evaluate the data shown in fig.5a of the main text, each triad was illuminated continuously at 488 nm for 100 s with the same intensity as for the corresponding photoconversion experiment.

Supporting References

1. Pu, S.-Z., Xiao, Q., Xu, J.-K., Shen, L., G.-Z. Li, Chen, B., Synthesis, photochromic kinetics, fluorescence and structure of a new 1,2-diarylcyclopentene. *Chin. J. Chem.* **24,** 463-467 (2006).

2. Yamamoto, S., Matsuda, K., Irie, M. Photochromism of Diarylethenes Linked by Hydrogen Bonds in the Single-Crystalline Phase. *Chem. Eur. J.* **9**, 4878-4886 (2003).

3. van Heerden, P. S., Bezuidenhoudt, B. C. B., Ferreira, D. Improved synthesis for the rodenticides, diphenacoum and brodifacoum. *J. Chem. Soc., Perkin Trans.* **1***,* 1141-1146 (1997).

4. Lindner, S. M., Thelakkat, M. Fluorescent dye-labeled polymers carrying triphenylamine, styrene, or acrylate pendant groups. Macromol. *Chem. Phys*. **207**, 2084-2092 (2006).
